# *2023 MCADD patient and family education summit with providers*: meeting highlights, congruences and contradictions

**DOI:** 10.1186/s13023-025-04117-0

**Published:** 2026-04-09

**Authors:** Rani H. Singh, Saran R. Gurung, Aileen Kenneson, Hans C. Andersson

**Affiliations:** 1 Department of Human Genetics, Emory University School of Medicine, Atlanta, GA USA; 2 Tulane University Medical Center, Hayward Genetics Center, New Orleans, LA USA; 3 101 Woodruff Circle, 7th Floor Suite 7130, Atlanta, GA 30322 USA

**Keywords:** Medium chain acyl coA dehydrogenase deficiency, MCAD deficiency, Family, Community, Symposium

## Abstract

**Background:**

Medium chain acyl CoA dehydrogenase deficiency (MCADD) is a rare, autosomal recessive disorder of fatty acid β-oxidation, which is typically diagnosed via newborn screening in the United States. Nutrition management during times of wellness and emergency care during times of illness are necessary to optimize outcomes. While guidelines for care have been published, there is variability in management between clinics.

**Body:**

The *2023 MCADD Patient and Family Education Summit with Providers* was a global virtual summit of parents/carers of children with MCADD, adult patients, and healthcare providers to explore current trends and to identify unmet needs related to MCADD management. The summit included presentations by specialists and open discussions between patients, parents/carers and providers. We identified areas in which patients/carers and providers were in agreement (e.g., provision of emergency letters), and areas where there appeared to be discordance (e.g., provision of sick day protocols and use of home glucose monitors).

**Conclusion:**

The summit provided an innovative model for education and research, effectively engaging a global audience around patient-centered priorities and to advance research. Creating more opportunities for such educational and discussion forums could benefit both attendees and those involved in MCADD or other rare genetic disorders research, potentially improving patient outcomes.

## Introduction

Medium chain acyl CoA dehydrogenase deficiency (MCADD, OMIM # 201450) is a rare, autosomal recessive disorder of fatty acid β-oxidation [1] with an incidence of 1 in 15,000 to 1 in 20,000 live births in the United States [2]. Because early detection and treatment drastically reduces morbidity and mortality [3–6], MCADD is included in the United States Department of Health and Human Services’ (DHHS) Recommended Uniform Screening Panel (RUSP), a list of disorders that the Secretary of the DHHS recommends for states to include in their newborn screening (NBS) programs [7]. In the United States, every state and the District of Columbia include MCADD on their NBS panels (https://babysfirsttest.org).

Standard treatment for MCADD is medical nutrition therapy, which in times of good health includes the avoidance of periods of fasting, as well as avoidance of consumption of foods that contain medium chain fatty acids [8]. Other management strategies used by some healthcare providers include home glucose monitors, carnitine supplementation, and cornstarch at bedtime to reduce the risk of hypoglycemia [8, 9]. There is variability in accepted practice between clinics [10, 11].

Periods of fasting, such as those precipitated by illness, can lead to a metabolic crisis in individuals with MCADD. In such instances, emergency care focuses on administration of intravenous dextrose-containing fluids [9]. Emergency resources such as letters that patients/carers can give to emergency department personnel, sick day protocols, and medical alert bracelets and necklaces are important resources for patients/carers [12]. As individuals mature, additional risk factors arise such as dieting, competitive sports, surgery, pregnancy and delivery, and alcohol and drug consumption [13].

To explore current trends and to identify unmet needs related to MCADD management, we convened a global virtual summit of adult patients, parents/carers of children with MCADD, and healthcare providers managing MCADD. The *2023 MCADD Patient and Family Education Summit with Providers* was co-hosted by the Southeast Regional Genetics Network (SERN) and Emory University’s Medical Nutrition Therapy for Prevention (MNT4P) program (https://mnt4p.org) [14]. This report provides an overview of the summit and summarizes information shared by participants.

## Methods

The virtual summit offered registration to healthcare providers, patients, and families managing MCADD worldwide. Promotional materials were distributed through HRSA regional genetics networks, parents’ organizations, outpatient clinics, and the Emory University GNO Metab Listserv which includes all Genetic Metabolic Dietitians International (https://gmdi.org) group members as well as other practitioners who treat patients with inherited metabolic disorders (IMD) or who have an interest in the nutrition management of IMDs. To understand current MCADD management and needs, registrants were asked to complete a pre-summit eight-question online survey via Qualtrics.

The summit took place in February 2023, and consisted of four main components. The first part featured an overview presentation of MCADD, focusing on the biochemistry of MCADD and the pathophysiologic mechanisms of the disorder, NBS and its impact on outcomes, treatment and management of MCADD, and strategies for fostering healthy long-term outcomes. The second part was a presentation on nutrition management of MCADD, including results of the pre-summit survey, research updates (on NBS, management principles, carnitine supplementation, and lived experiences of patients and families), clinical pearls regarding acute management, and patient perspectives. The third part included an open discussion session during which participants could ask questions. Topics of discussion included MCADD and Covid-19, anti-nausea medication, recommendations for managing diarrhea, use of glucometers, recognition of hypoglycemia symptoms, challenges in rural hospitals, surgical and procedural recommendations, and cardiac evaluations. Finally, the fourth part included a patient/carer panel discussion focusing on experiences with diagnosis and ongoing management, advocacy, communication with primary care providers and educators, experiences in rural emergency departments, emotional and mental health, self-care, and health issues in adults with MCADD.

Throughout the summit, real-time polling questions were used to gather additional quantitative data regarding recommendations and usage of medical alert identifiers (jewelry such as bracelets or necklaces) and home glucose monitors.

## Results

The *2023 MCADD Patient and Family Education Summit with Providers* was conducted via online videoconference in February 2023, with 204 participants including adult patients with MCADD, parents/carers of individuals with MCADD, healthcare providers, and other stakeholders.

A total of 240 individuals from 20 countries registered for the event with the majority (86%) from the United States. Of those who registered, 133 completed the pre-summit survey (Table 1). The survey included an open-ended question soliciting proposed agenda topics for the summit. Ninety-six registrants provided comments that were identified in seven thematic areas: overview of MCADD (*n* = 21), clinician guidelines and best practices (*n* = 21), interests of caretakers and support strategies for family members with MCADD (*n* = 14), nutrition guideline updates (*n* = 14), research updates (*n* = 12), patient experiences and perspectives (*n* = 7), and specific dietary concerns (*n* = 7).

**Table 1:** Description of summit registrants who completed the pre-summit survey

	Registrants (*N* = 133)
Adult patient with MCADD	4%
Parent/Carer	40%
Healthcare Provider:	
Medical Doctor (MD) Registered Dietitian (RD) Nurse Practitioner (NP) Registered Nurse (RN)	2% 41% 1% 3%
Other/not stated	9%

In the pre-summit survey, healthcare providers and patients/carers were asked about various aspects of the management of MCADD (Table 2). For some topics, patient/carer and provider responses aligned each other. For example, both indicated that MD geneticists were a source of care for most patients. However, while 73% of providers indicated that registered dietitians were involved in patient care at their clinic, only 33% of patients/carers reported seeing a registered dietitian regularly. There was general agreement on core management strategies, including fasting time limitations, carnitine supplementation, and dietary fat limitation though the use of cornstarch at bedtime was reported infrequently by both groups.

**Table 2:** Registrant responses to pre-summit survey

	Healthcare providers (*n* = 67)		Patients/ carers (*n* = 51)
Which providers see patients with MCADD in your clinic? Check all that apply.		Whom do you or a family member see for your MCADD management? Check all that apply.	
MD Geneticist RD NP RN Other	90% 73% 37% 22% 15%	MD Geneticist Primary Care Provider Other MD Dietitian Nurse Other	92% 45% 39% 33% 6% 12%
What diet recommendations are provided in your clinic? Check all that apply.		What diet recommendations are you or a family member on? Check all that apply.	
Fasting time limitations Carnitine supplementation Limit dietary fat Cornstarch at bedtime Other	82% 52% 25% 12% 12%	Fasting time limitations Carnitine supplementation Limit dietary fat Cornstarch at bedtime Other	80% 45% 25% 6% 24%
Do you provide emergency letters to patients?		Do you or a family member have an emergency letter from your provider?	
Yes No	91% 9%	Yes No	92% 8%
Do you provide sick day protocols to patients?		Do you or a family member have a sick day protocol from your provider?	
Yes No	69% 31%	Yes No	33% 67%

Consensus existed among patients/carers and providers about the use of emergency letters (91% of providers issued them; 92% of patients/carers received them), but responses differed significantly regarding sick day protocols: 69% of providers reported issuing them, but only 33% of patients/carers indicated receipt.

During the summit, real-time poll questions were used to gather quantitative data on two areas of discussion: use of medical alert identifiers (bracelets or necklaces) and home glucose monitors (Table 3). Initially 44% of healthcare providers reported that they would recommend medical alert identifiers, increasing to 83% after discussion. Similarly, only 16% of patients/carers indicated current use of such medical alert identifiers, but after discussion rising to 97%, indicating willingness to use if recommended.

**Table 3:** Responses to real-time poll questions

Healthcare providers (*n* = 30)		Patients/carers (*n* = 48)	
**Medical Alert Tags**			
*Prior to discussion of topic during summit*			
Are you recommending the use of medical alert bracelet or necklace to notify medical personnel for your patients?		Are you currently using a medical alert bracelet or necklace to notify medical personnel?	
Yes No	44% 56%	Yes No	16% 84%
*After discussion of topic during summit*			
Would you recommend the use of medical alert bracelet or necklace for your patients?		Would you use a medical alert bracelet or necklace if recommended?	
Yes No	83% 17%	Yes No	97% 3%
**Home Glucose Monitors**			
Do you see the benefits in prescribing a home glucose monitor to your patients?		Has your provider prescribed you a home glucose monitor?	
Yes No	56% 44%	Yes No	21% 79%
Have you prescribed a home glucose monitor to your patients?		Are you currently using a home glucose monitor?	
Yes No	33% 67%	Yes No	26% 74%

## Discussion

The *2023 MCADD Patient and Family Education Summit with Providers* offered vital educational and networking opportunities for patients, carers, and healthcare providers. It also highlighted critical areas of disconnect in MCADD management both between healthcare providers and their patients, while also revealing critical gaps in MCADD management practices. Responses to pre-summit surveys and poll questions during the summit highlighted substantial variability in care methods across clinical settings. For instance, guidelines suggest that the intake of dietary fat should be limited to 30–35% of kcals in children and adults [12, 15], with an avoidance of medium chain fats [15], but only 25% of healthcare providers indicated that they recommended such limitations.

Similarly, use of cornstarch at bedtime to prevent hypoglycemia was reported by only 12% of providers, with just 6% of patients/carers indicating its use. While this practice was reported at higher frequencies in earlier studies [10, 11], there does not appear to be any systematic investigation into its effectiveness specifically for MCADD. *GeneReviews* suggests avoidance of fasting which may require frequent feeding (every 2–3 h) in infancy, overnight feeding, a bedtime snack, or 2 g/kg of uncooked cornstarch for individuals with MCADD, though notes that cornstarch may not be necessary in the absence of intercurrent illness [1]. We did not ask providers under which conditions they recommended cornstarch use, so it remains unclear whether the low rates reflect a general shift away from routine use, or selective use during illness. Literature suggests that this practice may have been extrapolated from protocols used in glycogen storage disorders like GSD Ia, rather than from direct evidence in MCADD populations [16]. Earlier studies such as Solis & Singh (2002) and Potter et al. (2012) did not specify whether bedtime supplementation with cornstarch was for routine management or during illness [10, 11]. During the summit, providers indicated a preference for use of bedtime snacks that are high in complex carbohydrates rather than cornstarch, citing improved nutritional value and integration into daily energy needs.

There was strong agreement on the importance of fasting time limitations, reported by 82% of providers and 80% of patients/carers. While our survey did not ask respondents to specify exact fasting durations, one recent chart review found that published guidelines are commonly used as a starting point for clinical decision-making [17]. However, a Canadian multicenter review reported marked variation and lack of harmonization in fasting recommendations across clinics, despite shared diagnostic criteria and similar patient populations [18]. These guidelines, summarized in Table 4, are based on expert consensus and retrospective data, rather than prospective clinical trials. As such, they appear to serve as best-practice recommendations in the absence of stronger evidence. Further research is needed to determine whether healthcare providers consistently adhere to these fasting time guidelines across clinical settings.

**Table 4:** Published fasting time recommendations for children with MCADD, excluding periods of illness or metabolic stress

Derks (2007) [19]		GMDI (2008) [12]		Feillet (2012) [15]	
Age	Hours	Age	Hours	Age	Hours
6–12 months	8	Birth – 4 months	4	Birth – 2–4 weeks	3–4
1–2 years	10	5–12 months	Add one additional hour for each month of age	1–4 months	4–6
2 or more years	12			4–8 months 8–10 months 10–12 months 1–6 years > 6 years	6–8 8–10 10–12 12 < 14

Carnitine supplementation was another area of inconsistent practice. Only 45% of patients/carers reported using it and only half of providers recommended it. The necessity and efficacy of carnitine supplementation remain controversial [8, 9, 20], and there was a lack of understanding of the benefit of carnitine supplementation and the impact of carnitine deficiency by both groups. However, many providers noted during the summit that they monitored carnitine levels and only supplemented with carnitine when the blood concentrations were low. While this approach is supported by guidelines [1, 12, 15, 20, 21], practices varied widely across all clinics. We did not collect information about the dosage of carnitine used for supplementation. Dosing varied in the current literature with past and current sources recommending ranges from 20 to 100 mg/kg/day, divided into two or more doses [3, 11, 22, 23]. High doses may cause side effects, such as a fish-like odor [24].

There were two areas of discrepancy between healthcare provider perspectives and patient/carer experiences: sick day protocols and home glucose monitors. While 69% of healthcare providers claimed to offer sick day protocols, only 33% of patients/carers recalled receiving them. In contrast, both groups reported similar rates of emergency letter provision (91% of providers and 92% of patients/carers). The gap in sick day protocol recollection could reflect issues with patient retention, distribution, or protocol clarity. It is not clear which healthcare provider gave the sick day protocol to the patients, or whether the type of provider would influence whether the patient/carer recalled receiving them. While majority of healthcare provider respondents were RDs, it is important to note that in many clinical settings—including ours—RDs often provide these protocols on behalf of the medical team. This practice reflects coordinated care rather than independent prescriptive authority. We did not ask respondents to specify who authored versus who distributed these protocols, which limits our interpretation.

Regarding glucose monitoring, there are no published guidelines or recommendations for routine use of home glucometers. Despite our finding that 56% of providers supported glucometer use, only 33% medically recommended monitors, and only 25% of patients/carers reported using one. Moreover, sole reliance on glucometer readings may obscure early clinical signs of metabolic decompensation—such as lethargy, vomiting, or hypotonia—that can precede or occur in the absence of hypoglycemia. Concerns about over-medicalization due to the frequent use of glucometers [1, 9] were acknowledged during provider discussions. Discussion also focused on future research needs such as benefits, risks, prescription barriers, insurance challenges, implementation challenges, and patient education associated with glucose monitoring methods.

Participants expressed interest in several additional topics, including transitioning to adult healthcare, pregnancy planning, weight loss, exercise tolerance and emerging therapies. Family members and carers voiced the need to understand how to manage care transitioning as patients age and develop self-advocacy skills. Finally, there was widespread support for medical alert systems, such as MedicAlert^®^ bracelets (https://www.medicalert.org). Other medical alert systems include smartphones, in which health information can also be stored in the smartphone emergency medical identification (SEMID) applications, making information available to medical personnel and first responders. However, smartphones are not always available to or accessed by medical personnel in emergency situations [25, 26]. Regardless of which tool is chosen, medical alert systems should clearly state “MCADD,” “at risk of hypoglycemia,” and “give glucose” [12].

As already described, interpretation of the responses is limited by the survey format which lacked nuance regarding ill versus well states, fasting specifics and geographic variation. Additionally, clinic representation was uneven. Interpretation of the results is also limited by the wording of some of the questions which lacked clarity. For example, in asking which providers patients saw in the clinic, we did not specify whether we meant seen at the first visit, ever seen, or usually seen. These results were collected as a snapshot of current practices as perceived by both patients/carers and healthcare providers, and to inform future research in this area. In conclusion, the *2023 MCADD Patient and Family Education Summit with Providers* provided an innovative hybrid model for education and research, effectively engaging global audience around patient-centered priorities and to advance research. The relatively high attendance underscored the interest in this rare disease and the value of collaborative virtual platform. Moving forward, creating more opportunities for such educational and discussion forums could benefit both attendees and those involved in MCADD or other rare genetic disorders research and patient outcomes.

Providers should work toward evidence-based and consensus-driven protocols to establish standards of care for rare genetic disorders, harmonizing practices to better meet patient needs. In order to best meet these needs, the input of patients and carers (as demonstrated by other models such as the Canadian Inherited Metabolic Diseases Research Network (CIMDRN) [18]) is critical in both the collection of data to support an evidence-based process and the development of clinical practice guidelines. Providers should also consider regular reviews of protocols with patients and carers. Emphasis should be placed on reducing practice variation, enhancing access to key tools, and addressing priority topics in both clinical and research agendas.

## Data Availability

The data that support the findings of this study are available from the corresponding author upon reasonable request, subject to the institution’s data sharing policies.

## Abbreviations

CIMDRN: Canadian Inherited Metabolic Diseases Research Network
DHHS: Department of Health and Human Services
IMD: Inherited metabolic disorder
MCADD: Medium chain acyl CoA dehydrogenase deficiency
MNT4P: Medical Nutrition Therapy for Prevention
NBS: Newborn screening
RUSP: Recommended Uniform Screening Panel
SEMID: Smartphone emergency medical identification
SERN: Southeast Regional Genetics Network
